# Upfront Treatment of Pediatric High-Risk Neuroblastoma With Chemotherapy, Surgery, and Radiotherapy Combination: The CCCG-NB-2014 Protocol

**DOI:** 10.3389/fonc.2021.745794

**Published:** 2021-11-15

**Authors:** Dongdong Zhang, Natasha Mupeta Kaweme, Peng Duan, Youhong Dong, Xiaojun Yuan

**Affiliations:** ^1^ Department of Pediatric Hematology/Oncology, Xinhua Hospital Affiliated to Shanghai Jiao Tong University School of Medicine, Shanghai, China; ^2^ Department of Oncology, Xiangyang No. 1 People’s Hospital, Hubei University of Medicine, Xiangyang, China; ^3^ Department of Hematology, Zhongnan Hospital Affiliated to Wuhan University, Wuhan, China; ^4^ Department of Obstetrics and Gynaecology, Xiangyang No. 1 People’s Hospital, Hubei University of Medicine, Xiangyang, China

**Keywords:** neuroblastoma, high risk, CCCG-NB-2014, overall survival, N-myc

## Abstract

**Purpose:**

The Chinese Children’s Cancer Group developed the CCCG-NB-2014 study to formulate optimal treatment strategies for high-risk (HR) neuroblastoma (NB). The safety and efficacy of this protocol were evaluated.

**Method:**

Patients with newly diagnosed neuroblastoma and defined as HR according to the Children’s Oncology Group study were included. They were treated with a combination of chemotherapy, surgery, and radiotherapy. The treatment-related toxicities, response rate, 3-year progression-free survival (PFS), and overall survival (OS) were analyzed.

**Results:**

Of 159 patients enrolled between 2014 and 2018, 80 were eligible, including 19 girls and 61 boys, with a median age of 3.9 years (range 0.9–11). After a median follow-up of 24 months (range 3–40), the median OS was 31.8 months, and 3-year OS was 83.8%. In multivariate analyses, the OS was affected by N-MYC amplification (hazard ratio 0.212, 95% confidence interval (CI) 0.049–0.910; *p* = 0.037) and giant tumor mass (hazard ratio 0.197, 95% CI 0.071–0.552; *p* = 0.002). The median 3-year PFS was 25.8 months, and 3-year PFS was 57.5%. The univariate analysis showed that only the giant tumor mass was associated with the outcome. Of the 13 deaths, 11 died from the rapid progression of the disease and two from treatment-related toxicities. The most common adverse reaction was chemotherapy-induced hematological toxicity.

**Conclusion:**

The PFS and OS reported in our study were similar to Western countries. The CCCG-NB-2014 protocol proved to be an efficient regimen with tolerable side-effect for the treatment of pediatric HR-NB.

## Introduction

Neuroblastoma (NB) is the most prevalent malignant extracranial solid tumor in children and may be considered to originate from the adrenal medulla or paravertebral sympathetic nervous system. NB generally occurs in children under the age of 15, with a median diagnosis age of 17 months and an incidence of 10.2 per million (1, 2). The disease has a variable clinical presentation due to its marked tumor heterogeneity. NB could spontaneously regress with no symptoms; adversely, it can also progress rapidly with local invasion and metastasis. With the efforts of international multicenter cooperation groups, the 5-year-related survival rate of NB was substantially improved between 1975 and 2009, from 54% to 79% (3).

NB has a highly variable prognosis; therefore, developing a practical risk-classification algorithm to guide the treatment was necessary. Age less than 18 months was defined as a favorable prognosis as patients between 12 and 18 months of age achieved a longer survival (4). Tumor stage formulated by the International Neuroblastoma Staging System (INSS) could indirectly reflect tumor burden and the underlying tumor biology (5). Moreover, N-MYC amplification and tumor histopathological grade were biological prognostic factors (6, 7). On the basis of age at the initial diagnosis, the International Neuroblastoma Pathology Classification (INPC), INSS and N-MYC amplification status, and the Chinese Children’s Cancer Group (CCCG) developed a risk-stratification system and classified NB into three categories: low risk (LR), intermediate risk (IR), and high risk (HR).

The HR-NB had a relatively low initial response rate and poor prognosis than the LR-NB and IR-NB, with only 20%–35% survival rate (8–10). The main treatment method for HR-NB was intensive chemotherapy and surgical resection in the induction phase, external beam radiotherapy (XRT) and autologous bone marrow transplantation (ABMT) in the consolidation phase, and cis-retinoic acid (Cis-RA) in the maintenance phase. The ABMT could improve 5-year event-free survival (EFS) in patients with HR-NB (8); however, the treatment is a challenge for clinicians in many developing countries because of limited resources. Studies have shown that intensive chemotherapy could also improve clinical outcomes. Patients receiving chemotherapy without ABMT who achieved completed remission (CR) and very good partial remission (VGPR) had a better EFS than those who achieved partial remission or lower (8). However, 15%–20% of the patients with HR-NB were refractory to induction chemotherapy; approximately 50% of the patients with HR-NB either progress during the induction period or recurrence following ABMT (8, 11, 12). Therefore, developing a potent induction regimen was important for patients with HR-NB.

In 1994, the Memorial Sloan-Kettering Cancer Center (MSKCC) introduced a basic induction therapy, including high-dose cyclophosphamide combined with doxorubicin and vincristine. This protocol could help a majority of patients with HR-NB achieve CR or VGPR but with significant hematological and mucosal toxicities (13–15). From 1999 to 2010, the CCG-3891 study conducted by the Children’s Oncology Group (COG) showed dose-intensive induction and consolidation could improve clinical outcomes, for example, chemotherapy regimen including high-dose cyclophosphamide or ifosfamide, cisplatin, doxorubicin, and etoposide (8, 12). The CCG-3891 induction chemotherapy incorporated four agents with nonresistance mechanisms of cytotoxicity absence of vincristine and increase the chemotherapy cycle to eight cycles, subsequently with the high incidence of grades 3 and 4 adverse effects. A phase II clinical trial (COG-ANBL02P1) in 2010 showed that topotecan combined with cyclophosphamide could decrease the rate of infectious complications, improve the response rate, and delay the progression time in patients with recurrent NB (16). Therefore, COG added two cycles of topotecan and cyclophosphamide combined with four cycles of N7 chemotherapy for the pilot induction of the newly diagnosed HR-NB in 2011 (COG-2011 pilot study); this protocol is confirmed to be safe and feasible for patients with HR-NB (17). The CCCG formulated the CCCG-NB-2014 protocol to explore the optimum induction therapy for NB on the basis of the MSKCC regimen, COG-ANBL02P1 study, and COG-2011 pilot study in 2014. We analyzed the response rate, toxicities, survival rate, and prognostic factors in patients with newly diagnosed HR-NB who were treated according to the CCCG-NB-2014 protocol in this prospective study.

## Patients and Methods

### Patient Eligibility and Study Design

Patients below 18 years who were newly diagnosed with NB, and previously untreated, and with adequate performance and organ functions were enrolled between October 2014 and December 2018 in our study. The diagnosis of NB was based on tissue pathology or evidence of bone marrow involvement with increased urine vanillylmandelic acid (VMA) and serum nonspecific esterase (NSE) metabolites (5). Clinical staging was based on INNS and International Neuroblastoma Risk Group staging system (5, 18). The risk stratification was referred to COG (11, 19, 20). Only patients defined as HR were eligible. The detailed inclusion criteria are listed in **Table 1**.

**Table 1:** Definition of high-risk neuroblastoma according to the Children’s Oncology Group study.

Risk group	Age (year)	INPC grade	INSS grade	N-MYC
High risk	>1	UFH	2	Positive
	>1	UFH	3	Negative
	>1	FH	3	Positive
	>1.5	UFH or FH	4	Positive or negative
	<1	UFH or FH	3 or 4	Positive
	Any age	UFH or FH	4s	Positive

INPC, International Neuroblastoma Pathology Classification; INSS, International Neuroblastoma Staging System; FH, favorable histologic type; UFH, unfavorable histologic type.

Normally, patients underwent surgery and peripheral blood stem cell (PBSC) collection after four cycles of induction chemotherapy; subsequently, with four cycles of consolidation chemotherapy and radiotherapy, cis-retinoic acid (13-cis-RA) was used for maintenance therapy at the last stage. ABMT was recommended to be performed twice on the patients, before and after radiotherapy. In some cases, the operation was carried out ahead of schedule when clinicians needed the tumor tissue for a pathological investigation to confirm the diagnosis. In contrast, patients with giant tumor mass may receive more than four cycles of preoperative chemotherapy.

This study was approved by the Ethics and Scientific Committee of Xinhua Hospital Affiliated to Shanghai Jiao Tong University School of Medicine with approval number SHXH2021038 and was performed following Good Clinical Practice Guidelines and the Helsinki Declaration. All patients gave written informed consent before enrollment. All data were collected from the electronic medical record of Xinhua Hospital Affiliated to Shanghai Jiao Tong University School of Medicine.

### Treatment Response Evaluation

Treatment response was evaluated by detecting NSE and VMA levels, changes in primary lesions and metastases, and assessment of the N-MYC copy number and the minimal residual disease (MRD). The scan obtained from magnetic resonance imaging or positron emission tomography was used for imaging examinations. Fluorescence *in situ* hybridization was for cytogenetic assessment. Bone marrow aspiration and immunotyping were performed to evaluate the MRD. Therapeutic evaluation was based on the International Neuroblastoma Response Criteria and divided into imaging remission and metabolic remission, including complete remission (CR), very good partial remission (VGPR), partial remission (PR), mixed response (MR), no response (NR), and progressive disease (PD) (5, 21).

### Toxicity

Patients were examined by hematological and biochemical tests and electrocardiogram and echocardiogram before every cycle of chemotherapy. A hearing test must be performed before a cisplatin-based regimen. A diuretic renogram should be done to assess the renal function due to the use of high dose of cyclophosphamide. If two consecutive four-degree myelosuppressive events were observed after chemotherapy and the hemogram did not return to normal more than 1 week after supportive therapy, the drug dose must be modified in the next cycle of chemotherapy. The National Cancer Institute Common Terminology Criteria for Adverse Events version 4.0 was referred to as grade adverse reactions (22).

### Statistical Analysis

A univariate analysis was performed for age, sex, N-MYC status, skeletal metastases, bone marrow invasion, and tumor mass <10 cm to estimate the prognostic risk factors of the 3-year PFS by analysis of variance test. A multivariate COX regression analysis was performed to assess the effect of prognostic risk factor on OS. OS and PFS were estimated by the Kaplan-Meier curve. Statistical significance was determined by a *p*-value <0.05. IBM SPSS Statistic 24 software was used for statistical analysis.

## Results

### Patient Characteristic

**Figure 1:**
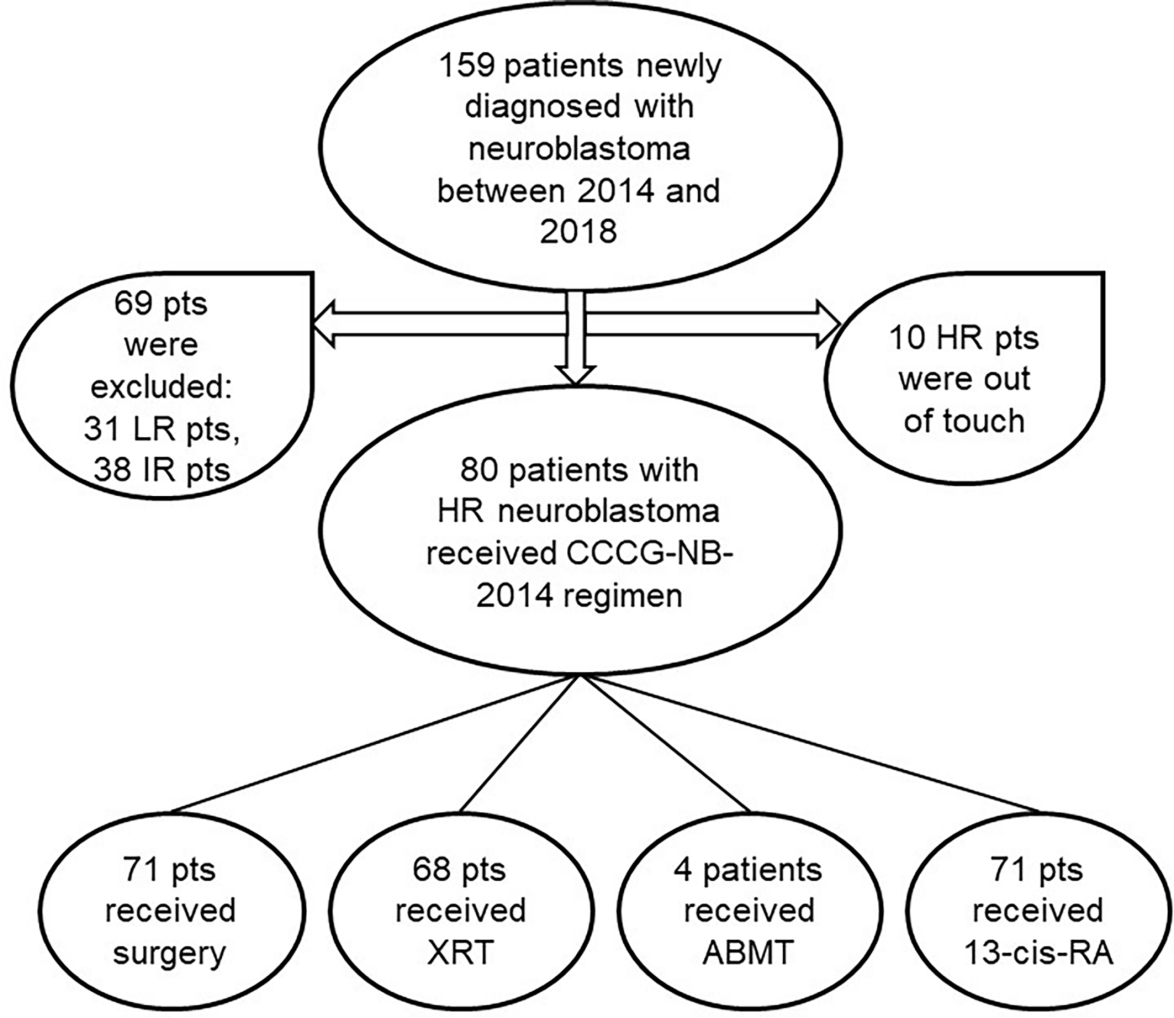
Flow diagram of patients in the study. pts, patients; LR/IR/HR, low/intermediate/high-risk; XRT, external beam radiotherapy; ABMT, autologous bone marrow transplantation; Cis-RA, cis-retinoic acid.

From October 2014 to December 2018, 159 patients newly diagnosed with NB were enrolled, including 31 LR, 38 IR, and 90 HR. The patients with HR could not be contacted after discharge from the hospital. Thus, 80 patients were eligible for our study (**Figure 1**), whose clinical and biological characteristics are summarized in **Table 2**. As shown in the table, HR-NB occurs at the median age of 3.9, mostly after 1.5 years of age (90%) and mainly in boys (68.86%). Adrenal medulla or abdomen was still the common primary site (82.5%). The common metastatic sites were bone marrow (44%) and bone (46%). Almost 97.5% of the patients had an elevated serum NSE level, but only 51.25% of the patients had urine VMA elevation and less than 57.5% of the patients had N-MYC amplification.

**Table 2:** Patient characteristics of study cohort.

Characteristic	No. (%)
Age at diagnosis (years)	
<1.5	8 (10.00)
>1.5	72 (90.00)
Median (range)	3.9 (0.9–11)
Gender	
Male	61 (68.86)
Female	19 (31.14)
Primary tumor site	
Adrenal or abdominal	66 (82.50)
Thoracic	9 (11.25)
Neck and other sites	5 (6.25)
Metastatic site at diagnosis	
Bone marrow	44 (55.00)
Bone	46 (57.50)
Lymph node	30 (37.50)
Other organs (liver, brain, lung)	25 (31.25)
Serum NSE at diagnosis	
Above normal	78 (97.50)
Normal or unknown	2 (2.50)
Urine VMA at diagnosis	
Above normal	41 (51.25)
Normal or unknown	39 (48.75)
N-MYC	
Positive	16 (20.00)
Negative	46 (57.50)
Unknown	18 (22.50)
Tumor mass >10 cm	
Positive	8 (11.25)
Negative	71 (88.75)

Data are presented as median (range) for continuous variables and number (%) for categorical variables. VMA, vanillylmandelic acid; NSE, nonspecific esterase; LOH, loss of heterozygosity.

### Treatment

**Figure 2:**
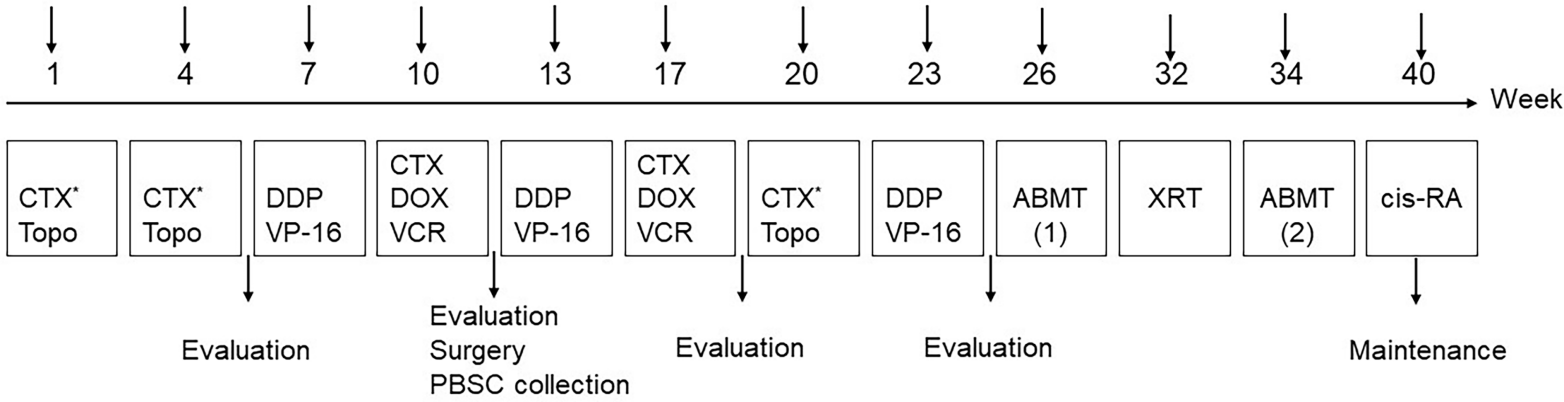
Treatment schedule. PBSC, peripheral blood stem cells; XRT, external beam radiotherapy; 13-cis-RA: cis-retinoic acid.

The detailed treatment schedules are summarized in **Figure 2**. All the patients received eight cycles of chemotherapy (**Table 3**). The efficacy was evaluated after every two cycles of chemotherapy. Carboplatin could be considered if children suffered from hearing impairment after treatment with a cisplatin-containing regimen; doxorubicin might be replaced by liposomal doxorubicin in the case of cardiac dysfunction. Generally, surgery and PBSC collection were performed after the fourth chemotherapy. ABMT was performed twice, one before and one after radiotherapy. Cis-RA was selected for the maintenance treatment for 6 months, with a routine dose of 160 mg/m^2^, administrated orally for 14 consecutive days each month. Trimethoprim-sulfamethoxazole was given for three consecutive days per week during treatment. Patients were requested to countercheck and followed up every 2-month after the end of treatment.

**Table 3:** CCCG-NB-2014 protocol.

Agents	Dose	Route	Time
CTX^*^+Topo			
Cyclophosphamide^*^	400 mg/m^2^ (<12 kg: 13.3 mg/kg)	IV	D1-5
Topotecan	1.2 mg/m^2^	IV	D1-5
*Irinotecan*	*120* *mg/m^2^*		*D1-3*
**DDP+VP-16**			
Cisplatin	50 mg/m^2^ (<12 kg: 1.67 mg/kg)	IV	D1-4
*Carboplatin*	*560* *mg/m^2^ (<12* *kg: 18* *mg/kg)*		*D1*
Etoposide	200 mg/m^2^ (<12 kg: 6.67 mg/kg)	IV	D1-3
**CTX+DOX+VCR**			
Cyclophosphamide	1,200 mg/m^2^ (<12 kg: 60 mg/kg)	IV	D1-2
Mesna	420 mg/m^2^ (0, 4, and 8 h after CTX injection)	Push	D1-2
Doxorubicin	25 mg/m^2^ (<12 kg: 0.83 mg/kg)	IV	D1-3
*Liposomal doxorubicin*	*20* *mg/m^2^*		*D1-3*
Vincristine	0.017 mg/kg (<12 months)	Push	D1-3
	0.67 mg/m^2^ (<12 months and 12 kg)		D1-3
	0.022 mg/kg (>12 months, below 12 kg)		D1-3

### Response and Outcome

The response rate is shown in **Table 4**. After four cycles of chemotherapy, 19 patients (21.25%) showed CR, 24 patients (30%) showed VGPR, and 17 patients (21.25%) showed PR. The overall objective remission rate (ORR; CR+VGPR+PR) was 60/80 (75%). Two patients died after four cycles of chemotherapy. After eight cycles of chemotherapy, the ORR was 61/78 (78.25%), of which the CR was 47.44% (37/78).

**Table 4:** Induction treatment response after four- and eight-cycle chemotherapy.

Treatment response	After 4 cycles [No. (%)]	After 8 cycles [No. (%)]
CR	19 (23.75)	37 (47.44)
VGPR	24 (30.00)	16 (20.51)
PR	17 (21.25)	8 (10.25)
MR	7 (8.75)	–
NR	5 (6.25)	–
PD	6 (7.50)	12 (15.38)
Death	2 (2.50)	5 (6.41)

Primary tumor resection was adopted before chemotherapy in patients evaluated as CR in the “four-cycle” group. CR, complete remission; VGPR, very good partial remission; PR, partial remission; MR, mixed response; NR, no response; PD, progressive disease.

**Figure 3:**
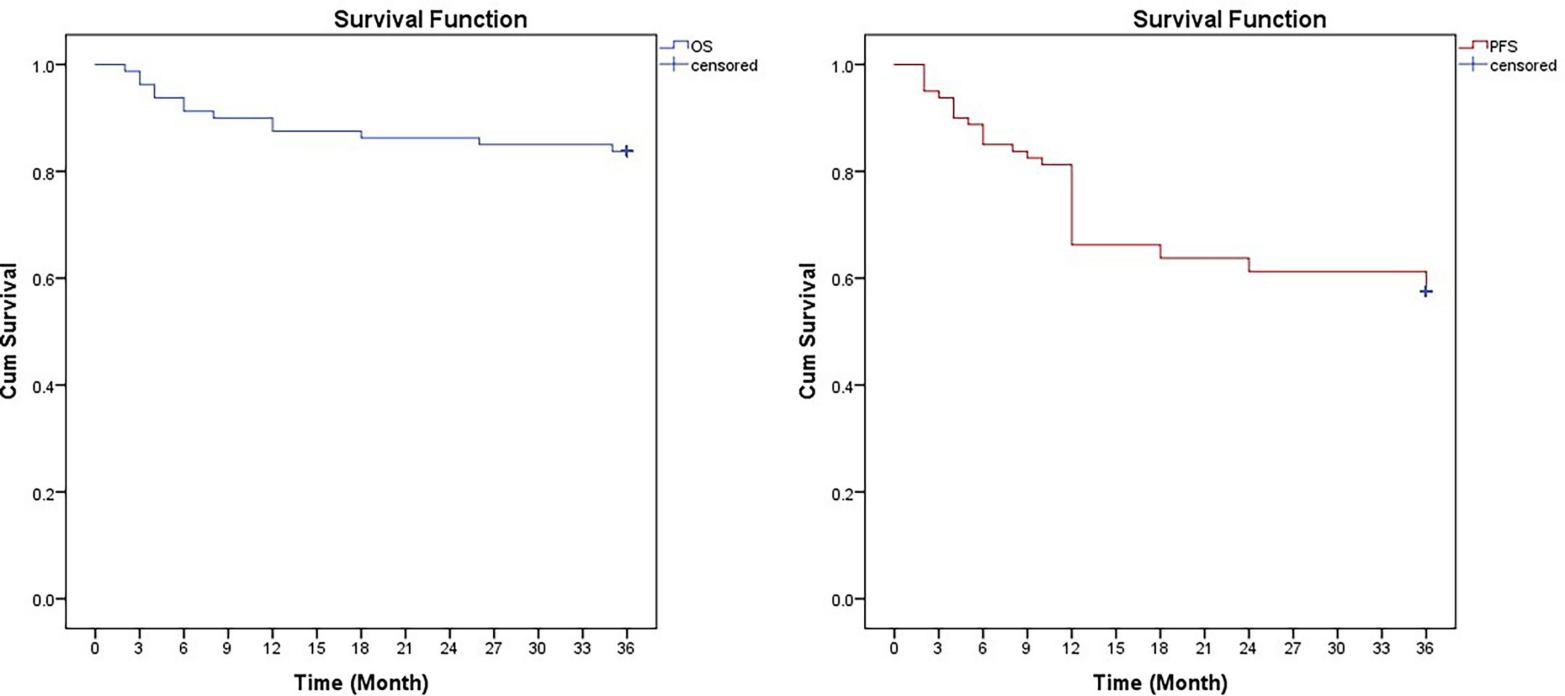
Kaplan-Meier curves for overall survival (OS) and progression-free survival (FPS) in 80 children with high-risk neuroblastoma treated with CCCG-NB-2014.

At a median follow-up of 24 months (range 3–40 months), 13 patients died due to disease progression. Two patients with skull and orbital metastasis experienced rapid progression and died in 3 months. One patient died of hemophagocytic syndrome. One patient died due to giant tumor rupture and massive blood loss in the short term after four cycles of chemotherapy. Three patients died of severe infection, one of whom died after two cycles of chemotherapy. Six patients died of widespread metastasis and secondary multiple organ failure. The 3-year PFS was 57.5% ± 4.2%, and the median PFS was 25.8 months. The 3-year OS was 83.8% ± 3.1%, and the median OS was 31.8 months (**Figure 3**).

### Adverse Events

All patients were assessed, and grades 3 and 4 adverse events were recorded. The toxicity or tolerability is listed in **Table 5**. Hematological toxicity was the most common treatment-related toxicity. Twenty-four patients experienced febrile neutropenia, 13 of whom showed documented infection, including 11 bacterial infections and two fungal infections. About 20% of the patients required red blood cell transfusion and platelet transfusion. Four patients received the modified regimen (75% dose) because of at least two four-degree myelosuppressive events. The incidence of hearing impairment was 22.5% (18/80) in our study. The common side effect included hepatic and renal dysfunction, cardiac dysfunction, mucositis, vomit, diarrhea, and electrolyte disturbances. Two patients were infected with B19 parvovirus, resulting in severe anemia and acquired aplastic anemia. The rare treatment-related toxicity was secondary acute myeloid leukemia (sAML). One patient was diagnosed with sAML 3 years after the end of treatment; whereas, another was diagnosed 6 years later.

**Table 5:** Treatment-related toxicities after induction therapy.

Toxicity	No. (%)
Febrile neutropenia	24 (30.00)
Documented infection	13 (16.25)
Red blood cell transfusion	16 (20.00)
Platelet transfusion	15 (18.75)
Hearing impairment	18 (22.50)
Elevated liver enzymes	12 (15.00)
Renal dysfunction	7 (8.75)
Cardiac dysfunction	5 (6.25)
Mucositis	3 (3.75)
Vomit, diarrhea, electrolyte disturbances	6 (7.50)
Coagulation disorders	9 (11.25)
Dose modifications	4 (5.00)
B19 virus infection	2 (2.50)
Secondary acute myeloid leukemia	2 (2.50)

### Prognostic Factor

A univariate analysis of the 3-year PFS showed that only tumor mass >10 cm was the predictor (*p* = 0.025) (**Table 6**). A multivariate analysis of the 3-year OS, N-MYC amplification (*p* = 0.037), and tumor mass >10 cm (*p* = 0.002) achieved statistical significance and was identified as predictive (**Table 7**). We did not analyze the relationship between ABMT and PFS/OS because only four patients received ABMT in our study.

**Table 6:** Univariate analysis of prognostic factors for 3-year PFS in 80 patients.

Characteristic	Univariate analysis	95% CI of 3-year EFS	*p*-value
Age			
>1	23.80 ± 12.62	20.79–26.81	0.385
<1	20.70 ± 14.52	9.61–30.39	
Sex			
Male	22.87 ± 12.97	19.55–26.19	0.572
Female	24.79 ± 12.60	18.71–30.87	
N-MYC amplification			
Positive	20.91 ± 12.81	15.23–26.59	0.303
Negative	24.24 ± 12.83	20.87–27.62	
Skeletal metastases			
Positive	22.96 ± 12.55	19.43–26.49	0.739
Negative	23.97 ± 13.52	18.82–29.11	
Bone marrow invasion			
Positive	21.88 ± 12.99	17.78–25.98	0.304
Negative	24.85 ± 12.65	20.74–28.95	
Tumor mass >10 cm			
Positive	14.33 ± 13.82	3.7–24.96	0.025
Negative	24.46 ± 12.34	21.54–27.39	

**Table 7:** Multivariate analyses of prognostic factors for overall survival.

	Hazard ratio (95% CI)	*p*-value
Age >1	2.540 (0.826–7.812)	0.104
N-MYC amplification	0.212 (0.049–0.910)	0.037
Tumor mass >10 cm	0.197 (0.071–0.552)	0.002
Bone marrow invasion	1.807 (0.888–3.678)	0.103
Skeletal metastases	0.495 (0.203–1.203)	0.121

## Discussion

NB accounted for only 7% of all pediatric malignant tumor, but the observed 5-year mortality was 21% (3). NB had a high tumor heterogeneity; this unique characteristic was obvious in HR-NB, including variable clinical presentation and clinical outcome (23). Studies showed that early response in HR-NB was a predicator of better clinical outcome (24, 25). Furthermore, an increase in dose intensity of induction chemotherapy could improve the initial response rate (26). However, with the escalation in dose intensity, the incidence of adverse reactions also increased. For this reason, we initiated the CCCG-NB-2014 study to provide an optimal balance between efficacy and safety of the HR-NB treatment.

In this pilot study, the induction regimen was formulated on the basis of the MSKCC regimen and the ANBL02P1 study. Three cycles of topotecan plus cyclophosphamide (TOPO/CTX) combined with dose-intensive chemotherapy were introduced in this regimen. Two cycles of TOPO/CTX were performed at the beginning for induction and one cycle at the end for consolidation preparation. The pharmacokinetically guided topotecan dose was 1.2 mg/m^2^ according to the COG pilot study (17).

The most common adverse reaction was hematological toxicity, about 30% of the patients developed myelosuppression and were hospitalized, 13% patients were infected and needed positive anti-infection treatment, and about 20% patients needed transfusion. Febrile neutropenia and significant mucositis rarely occurred during the first two cycles of chemotherapy because when incorporated with topotecan, the cyclophosphamide dose was reduced, resulting in a shortened hospital stay. TOPO/CTX treatment-related toxicities were tolerable and manageable in our study, which was similar to that of the previous study (16) and further indicated that the first two cycles of topotecan plus cyclophosphamide were feasible for the initial induction. Hearing impairment was another adverse reaction that required special attention, with 22.5% of the patients developing this symptom after cisplatin-containing chemotherapy. In general, we considered carboplatin, which had less ototoxicity, in such a case. Extension of duration of cisplatin infusion was confirmed to be invalid (27). The intravenous administration of sodium thiosulfate 6 h after the discontinuation of cisplatin could be helpful in decreasing ototoxicity (28). Two patients, who experienced B19 virus infection possibly due to hypoimmunity after chemotherapy, presented with refractory anemia, and subsequent aplastic anemia. This is an indication that we also need to pay attention to virus infections during the induction phase. Secondary malignancy was a rare long-time side effect. The diagnosis of sAML 3 years after the end of treatment in two patients could be related to the mutagenic potential of alkylating agents (29), which may provide a rationale for reducing the number of cycles of dose-intensive chemotherapy and the necessity of ABMT. Only four patients underwent dose modification (reduction to 75%) after the development of two consecutive four-degree myelosuppressive events. Considering the limited resource and expensive fee, only four patients underwent ABMT in our study. On the whole, the regimen was manageable and safe.

Within the context of the induction regimen, the patients achieved an overall response rate of 78.25% after eight cycles of induction chemotherapy, with 68% patients showing CR or VGPR. The ORR in our study was similar to the response rate (84%) in the ANBL02P1 induction regimen, but we achieved a better CR and VGPR (68% *vs*. 48%) (16). In the COG pilot study, PBSC collection was started after two cycles of CTX/TOPO. The European Bone Marrow Transplant registry demonstrated that the CR of metastases before ABMT was a predictor of a better clinical outcome (30). Our result showed that 53.75% of the patients achieved CR or VGPR after four cycles of chemotherapy (**Table 4**); therefore, we harvested the PBSC after two cycles of CTX/TOPO and two cycles of dose-intensive chemotherapy. More importantly, the PBSCs were less likely contaminated by tumor cells. The multivariate analysis indicated that the 3-year OS correlated with the N-MYC amplification and tumor mass >10 cm and was unrelated to age, stage, and metastasis site. However, two patients with skull and orbital metastasis died in a short time. Our result and other studies suggested skull and orbital metastases may be associated with poor prognosis (31). However, a large number of patients are still needed for further verification.

The pretreatment risk stratification of patients was essential and important for clinicians. The limitation of our study was that the diploid DNA index and the chromosome variation including 1p or 11q loss of the heterozygosity status were not detected to the limited resources. This caused our 2014 HR stratification criteria to differ from the latest COG stratification and INPC criteria (32, 33). Previous studies showed that myeloablative therapy and ABMT were related to EFS (8); we did not perform correlation analysis due to the limited number of patients.

The novel therapies provided more choices for the treatment of HR-NB. ^131^I-labeled metaiodobenzylguanidine (^131^I-MIBG) combined with topotecan was found to be effective in improving EFS and OS in patients with primary refractory HR-NB (34). The ^131^I-MIBG might develop as the first-line consolidation therapy in the future (35). Anti-GD-2 immunotherapy combined with interleukin 2 and Cis-RA was confirmed to be successful in eradicating the MRD in maintenance phase (36) and could significantly improve the clinical outcome (37). Anti-GD-2 monoclonal antibody was recommended for the maintenance therapy by CCCG although it was unlisted in China. *ALK* somatic mutation or gene amplification was observed in 15% of the patients with NB and 30% with NB-derived cell lines. Blocking the ALK mutation resulted in the inhibition of NB cell viability. This may provide new clinical insights into patients with NB with *ALK* mutation or gene amplification (38, 39). Some other emerging therapies like Aurora kinase A inhibitor (40), programmed death-1 inhibitor, and chimeric antigen receptor T cell were also promising therapies under study (41, 42).

In conclusion, the CCCG-2014-NB protocol was efficient in improving the clinical outcome. The treatment-related toxicities were tolerable and manageable, and this regimen has the potential to be recommended as the upfront treatment for the newly diagnosed HR-NB.

## Data Availability Statement

The raw data supporting the conclusions of this article will be made available by the authors, without undue reservation.

## Ethics Statement

This study was approved by the Ethics and Scientific Committee of Xinhua Hospital Affiliated to Shanghai Jiaotong University School of Medicine and was performed according to the Good Clinical Practice Guidelines and the Helsinki Declaration. Written informed consent to participate in this study was provided by the participants’ legal guardian/next of kin.

## Author Contributions

XY provided the direction of the study. DZ collected and analyzed the data and wrote the manuscript. YD helped collect the data. NK and PD revised the manuscript. All authors contributed to the article and approved the submitted version.

## Funding

This work was supported by the Innovative Research Program of Xiangyang No. 1 People’s Hospital (Grant number: XYY2021Q02).

## Conflict of Interest

The authors declare that the research was conducted in the absence of any commercial or financial relationships that could be construed as a potential conflict of interest.

## Publisher’s Note

All claims expressed in this article are solely those of the authors and do not necessarily represent those of their affiliated organizations, or those of the publisher, the editors and the reviewers. Any product that may be evaluated in this article, or claim that may be made by its manufacturer, is not guaranteed or endorsed by the publisher.
